# Congenital malaria: Frequency and epidemiology in Colombia, 2009-2020

**DOI:** 10.1371/journal.pone.0263451

**Published:** 2022-02-18

**Authors:** Jaiberth Antonio Cardona-Arias, Jaime Carmona-Fonseca

**Affiliations:** 1 School of Microbiology, University of Antioquia, Medellín, Colombia; 2 “Grupo de Investigación César Uribe Piedrahíta”, Faculty of Medicine, University of Antioquia, Medellín, Colombia; Instituto Rene Rachou, BRAZIL

## Abstract

Congenital Malaria (CM) is an underestimated and under-researched problem in Colombia, despite its severe clinical, epidemiological, economic, and public health consequences. The objective was to determine the general frequency of CM, the specific frequency of CM by diagnostic test and plasmodial species, and identify its associated factors. A retrospective study was carried out using the records of 567 newborns. qPCR and Thick Blood Smear (TBS) were performed. The frequency of infection was determined with a 95% confidence interval. Associated factors were identified by non-parametric tests and odds ratios; the confusion was controlled with a logistic regression model. All cases corresponded to submicroscopic CM (negative with TBS and positive with PCR), and the frequency was 12.2% (95%CI = 9.4–14.9). The detection was statistically higher in the umbilical cord with 16,2% (95%CI = 12.4–19.9) versus peripheral blood of the newborn with 2.2% (95%CI = 0.7–4.9). CM was statistically higher in newborn whose mothers had malaria in the last year, gestational and placental malaria. The median birth weight in newborn infected with CM was lower compared to the one of healthy neonates. Because the control program in Colombia is based on TBS, it must be improved with the inclusion of other tests that allow the detection of submicroscopic CM. In addition, the program has other limitations such as do not have specific actions for pregnant women and have a passive surveillance system. These difficulties do not allow to show the magnitude of CM, its consequences on neonatal and infant health, constituting a serious problem of health injustice.

## Introduction

Malaria associated with pregnancy (MAP) is a serious clinical-epidemiological problem for 125 million women in the world every year, but the real impact of the infection in the infant population and the magnitude of Congenital Malaria (CM) is unknown [1]. For example, in Colombia in 2019, 74,409 malaria cases were reported, and only 0.6% (n = 455) were in pregnant women [2], which would wrongly reflect a low risk of CM.

CM is the infection of the gestational product during uterine life or birth (not from the bite of an infected mosquito or blood transfusion), by asexual forms of *Plasmodium* spp., based on its detection in the newborn’s umbilical or peripheral cord blood from twenty-four hours to seven days of life, regardless of clinical symptoms; However, this definition is a matter of debate and lacks a clear consensus [3].

CM can be asymptomatic or symptomatic, and in the latter case, it varies from a nonspecific clinical manifestations, similar to neonatal sepsis or STORCH (syphilis, toxoplasmosis, other, rubella, cytomegalovirus, herpes virus), to other severe diseases. A serial thick blood smear (TBS) is recommended in endemic areas to establish the diagnosis [4]. Untreated neonatal infection can lead to serious outcomes such as anemia, metabolic acidosis, jaundice, renal failure, coagulopathy, shock, coma, and death [5, 6]. Furthermore, CM is correlated with preterm delivery, low birth weight, and increased infant morbidity and mortality [7].

In epidemiological terms, CM frequency is highly heterogeneous between and within countries, with figures ranging between 0.3% and 37% [6, 8]. For example, an incidence of 42.4% [7] was found in 112 Indonesian mother-child pairs by TBS or PCR (Polymerase Chain Reaction); In Uganda, a cross-sectional study of 261 mother-newborn pairs using TBS from umbilical cord blood found an incidence of 6.1% [9]; in Niger, in 249 neonates where peripheral and cord blood were tested, the incidence was 26.5% [10]; and in Sudan, including positives in newborn peripheral or umbilical cord blood, it was 18.6% by TBS and 56.8% by qPCR (quantitative) [11].

Studies on CM also report high heterogeneity in its associated factors, some pointing to low birth weight, anemia, and preterm delivery [7]. In contrast, others indicate that no clinical features were associated with CM (birth weight, gestational age, APGAR, length at birth, temperature, anemia), reporting association only with maternal parasitaemia, pregnancies (higher in primigravidae), maternal age (higher in those under 19 years of age) and placental malaria [9]. Other studies found an association with maternal age and hemoglobin, but not with parity, educational level, use of a mosquito net, area of residence, or neonatal weight [11]. While other authors only reported an association with the use of mosquito net [10].

A meta-analysis on the epidemiology of CM and neonatal clinical malaria captured 22 investigations that included 28083 neonates, in whom the prevalence of CM was 33.7% (95%CI = 6.9–77.2) with no differences among the studies conducted in Africa or elsewhere [12]. However, another meta-analysis with 24 studies of high methodological quality and 8148 neonates reported differences with a prevalence of CM of 3.5% (95%CI = 2.3–4.6) in areas with stable transmission, while in those with unstable transmission it was 16.8% (95%CI = 8.0–25.6) [13].

In Colombia, several studies have reported a low frequency of CM with TBS but a high with PCR; the following figures highlight the high heterogeneity within these studies, and among them, depending on the type of diagnostic test: *a)* with TBS incidence of 2.7% (95%CI = 1.1–6.4) in 183 newborns whose mothers were diagnosed with malaria [14], and 1.8% (95%CI = 0.5–7.0) in 110 samples from umbilical cord [15], *b)* 2.4% (95%CI = 0.6–9.0) with TBS and 13.1% (95%CI = 7.4–22.1) with PCR, in 84 umbilical cord samples [16]; *c)* 0.0% with TBS and 27.0% (95%CI = 20.2–35.0) with PCR in 137 umbilical cord samples [17]; *d)* 0.1% (95%CI = 0.0–0.6) in 636 umbilical cord samples and 0.0% in peripheral blood from 600 neonates with TBS, but with PCR it was 3.0% (95%CI = 1.0–8.9) in 100 umbilical cord samples [18]; *e)* 0.0% with TBS and 29.2% (95%CI = 21.0–39.0) with PCR in 96 umbilical cord samples [19].

The data presented reveal several problems:

1. High heterogeneity in the magnitude of CM and its potentials associated factors, which warrants local studies.
2. Low levels of knowledge about the clinical and epidemiological effects of CM, and the little available evidence are concentrated in Africa, where almost all cases are caused by *P*. *falciparum*.
3. In Colombia, there are few studies on CM with high diversity in its frequency.
4. Lack of knowledge about the potentials factors associated with CM in Colombian populations.
5. A high proportion of submicroscopic CM cases (negative with TBS and positive with PCR) in Colombian studies, which indicates that the problem is underestimated given that the care and treatment guidelines in the country are based on TBS, ignoring the short-term and long-term effect of this class of infections on the child.

To remedy to some extent the problems described, this research was carried out to measure the general and submicroscopic frequency of CM, as well as the specific frequency by diagnostic test and plasmodial species; and to identify their associated factors in a northwestern region of Colombia in the period between 2009–2020.

## Methods

### Study location

The region has 34,848 km, with around 1,150,000 inhabitants by 2020, and is made up of the areas of Urabá Antioqueño, the upper basins of the Sinú and San Jorge rivers, and the Bajo Cauca Antioqueño. It has had a high incidence of malaria (annual parasite index> 25 per 1,000 exposed) since 1950. This region is made up of 25 municipalities, distributed in the departments of Antioquia (11 from Urabá and 10 from Bajo Cauca) and Córdoba (4 municipalities) [20].

### Study type and subjects

A retrospective study was carried out with data from 567 newborns captured in five prospective and cross-sectional research projects on malaria associated with pregnancy developed by the Research Group “Salud y Comunidad César Uribe Piedrahíta” (these projects gave rise to the four publications cited in references 15,16,17 and 19). Newborn were captured in the obstetric services of municipal hospitals in the endemic region of northwestern Colombia between 2009 and 2020.

DANE (in Spanish Departamento Administrativo Nacional de Estadísticas de Colombia) indicate that study zone has 1,114,000 inhabitants with about 5,500 pregnant women exposed to paludism. In the study area, the coverage of the prenatal control program is 94%, to which are added malaria control sites in remote areas for diagnosis and treatment of the disease. In this context the investigations were developed, in which did not carry out any specific activity to promote attendance at prenatal consultation or delivery at the local hospital, with the purpose that the population represents the usual dynamics of delivery assistance in the region, and to control possible selection biases. All the women in the investigations were recruited in one of the public hospitals, either in prenatal consultation or during labor, to determine the frequency of CM in pregnant women living in endemic areas. Through an invitation and brief explanation that a doctor made to each pregnant woman, if she agreed to participate, the informed consent was explained to later sign it.

The pregnant women who participated in these research projects met the following inclusion criteria: stable residents (at least one year before the beginning of each study), pregnant women in whom the two diagnostic tests (TBS and qPCR) were performed, apparently healthy as recorded in their clinical chart (without a diagnosis of diseases or infections, without malaria complications during pregnancy, without symptoms of malaria), with voluntary participation in the study and signing of informed consent (for those of legal age) or assent (for minors).

### Malaria diagnosis

Peripheral or cord blood samples were taken from each newborn on the day of delivery. In cases where the clinical condition of the pregnant woman or the institutional characteristics of the hospital made it possible, samples were also taken for the diagnosis of gestational malaria (maternal peripheral blood with the presence of parasites according to TBS or qPCR, at any time of their corresponding pregnancy) and placental malaria (placental blood with the presence of parasites according to TBS or qPCR). Each blood sample was examined with TBS and qPCR. Slides were made for microscopic diagnosis by TBS, and Whatman filter paper circles # 3 were impregnated for DNA extraction with the Saponin-Chelex method and molecular diagnosis by qPCR.

### Epidemiological survey and extraction of data from the clinical history

A form was applied to obtain the following data from the clinical history and the pregnant woman: healthcare coverage, fetal death, stillbirth, hemoglobin, age, number of pregnancies, abortions and deliveries, use of mosquito net, and history of malaria in the last year. The following variables of the newborn were also extracted from the clinical chart: weight, length, and head circumference at birth, 1- and 5-minutes APGAR scores (considered normal with values ≥7 and abnormal for lower values).

### Control of information biases

The work team was trained in the standardized procedure for collecting and processing clinical samples, and recollecting data from the survey and the clinical chart. An internal quality control system was applied in the diagnostic tests, employing the blind and independent analysis of all the positive samples and 10% of the negative ones. The qPCRs followed the manufacturer’s instructions. A logical verification was carried out (that all the variables had values in the allowed range) during the data entry.

### Statistical analysis

The categorical variables were described with frequencies, and the continuous variables were described with summary measures (S1 Data). The general frequency of CM (positive by TBS or PCR) and the specific frequency by type of diagnostic test and plasmodial species were calculated with their 95% confidence intervals, which were compared with the Z test. The CM was compared with the categorical variables using Pearson’s Chi-square test or Fisher’s Exact, and with the continuous variables using the Mann-Whitney U test, since the assumption of normality was not met by using the Kolmogorov-Smirnov with Lilliefors correction. The strength of the association was established with crude and adjusted odd ratios, with logistic regression to identify effect modification (confusion or interaction) variables. All analyzes were performed in SPSS 27.0 with a significance of 0.05.

### Ethical aspects

The guidelines of the Declaration of Helsinki and Resolution 8430 of Colombia for research with pregnant women were applied. The study was classified as minimal risk and was endorsed by the Ethics Committee of the SIU (*Sede de Investigación Universitaria*), University of Antioquia, Act 21-101-961. All pregnant women signed the consent (of legal age) or assent (under 18 years of age) informed, obtained in writing, with the signature of the pregnant woman, a witness (external to the research group) and the member of the health team who explained the content of the informed consent-assent. According to Colombian law, adolescents (over 10 years old) can sign the informed assent without requiring consent from parents or guardians, as was the case of the adolescent pregnant women included in this research (informed consent were not obtained from the parent/guardians of pregnant women aged 13–17 years). All the data used in this research were anonymized by someone outside the research team (health personnel who participated in the collection of the information), so that the authors of this manuscript had access to a database with codes (without name, or identification number, or other information that will show the identity of the pregnant women), to guarantee the confidentiality of the data.

## Results

### CM frequency

All CM cases were submicroscopic infections. The general frequency of CM was 12.2%, the detection rate in umblical blood is significantly higher compared to the newborn peripheral blood (16.2% Vs 2.2%. Test Z = 5.3. p<0.001). The frequency by species was different depending on the type of blood sample analyzed (Table 1).

**Table 1:** Frequency of congenital malaria according to the type of blood sample (peripheral or umbilical cord blood) and diagnostic test (thick blood smear or qPCR).

	Frequency % (n)	95% CI
**N Total = 567**		
Congenital malaria	12.2 (69)	9.4–14.9
**Newborn peripheral blood N = 234**		
Thick blood smear	0.0 (0)	--
qPCR	2.2 (5)	0.7–4.9
*P*. *vivax*	1.3 (3)	0.3–3.7
*P*. *falciparum*	0.9 (2)	0.1–3.1
**Umbilical cord blood N = 396**		
Thick blood smear	0.0 (0)	--
qPCR	16.2 (64)	12.4–19.9
*P*. *falciparum*	10.1 (40)	7.0–13.2
*P*. *vivax*	4.8 (19)	2.6–7.0
Mixed	1.3 (5)	0.4–2.9

**Note**. Both peripheral and cord blood were collected in 63 subjects. 58/63 negative in both samples, 5/63 negative in peripheral blood but positive in cord blood (2 *P*. *falciparum*, 2 *P*. *vivax*, 1 mixed malaria)

### Maternal characteristics

The highest proportion of mothers were affiliated to the subsidized healthcare insurance, were adolescents or young women; 32.8% were in their first pregnancy; only 15% were nulliparous; 14% have had an abortion; 30% had anemia during pregnancy, and 25.1% had a history of malaria (Table 2).

**Table 2:** Description of clinical variables and malaria history of the mothers.

**Variable**	**Categories**	**n**	**%**
Healthcare coverage	Subsidized	339	59.8
	None	15	2.6
	Contributory	2	0.4
	No data	211	37.2
Age group	Adolescents (13–19)	166	29.3
	Youth (20–24)	178	31.4
	Adults (>24)	211	37.2
	No data	12	2.1
Pregnancies	One	186	32.8
	Two	122	21.5
	Three	97	17.1
	Four or more	144	25.4
	No data	18	3.2
Deliveries	Nulliparous (0)	86	15.2
	Primiparous (1)	103	18.2
	Multiparous (2 or more)	194	34.2
	No data	184	32.5
**Binary variables**	**No. of records**	**n**	**%**
Abortions	381	55	14.4
Stillbirth	379	6	1.6
Gestational anemia (hemoglobin<11)	395	118	29.9
History of malaria (last year)	558	140	25.1
No use of mosquito net	388	138	35.6
Gestational malaria (in current pregnancy)	444	99	22.3
Placental malaria (in current pregnancy)	447	109	24.4

### Neonatal characteristics

Birth weight showed a mean of 3135.3g ± 452.5g, a median of 3100g, an interquartile range of 2850g-3400g, and a range between 1100g-5300g (N = 452); length, showed a mean of 49.5cm ± 4.1cm, a median of 50cm, an interquartile range of 49-51cm, and a range of 25-58cm (N = 285); the head circumference showed a mean of 33.8cm ± 1.8cm, a median of 34cm, an interquartile range of 33-34cm, and a range of 25-44cm (N = 285). The 5-minute APGAR score was low (<7) in 1.8% of the newborns (N = 279), and 6.4% (N = 452) of them had a low birth weight.

### Factors associated with CM

A statistical association of CM was found only with having had malaria in the last year and having a confirmed diagnosis of gestational and placental malaria (Table 3). Furthermore, the birth weight was statistically lower in newborns affected by CM who had a median of 3000g (interquartile range = 2740g-3300g), compared to healthy neonates who had a median of 3100g (interquartile range = 2890–3420) (Mann-Whitney U test p = 0.025).

**Table 3:** Comparison of the general frequency of congenital malaria, and specific in the newborn’s umbilical cord and peripheral blood; according to maternal and neonatal variables.

Maternal and neonatal variables	Categories	Frequency % (n)^a^		
		General N = 567	Umbilical cord N = 396	Peripheral blood N = 234
Healthcare coverage	None	6.7 (1)	0.0 (0)	6.7 (1)
	Subsidized	2.9 (10)	3.3 (6)	1.9 (4)
Age group	Adolescents and young people (13–24)	12.8 (44)	16.7 (42)	1.5 (2)
	Adults (>24)	11.8 (25)	15.3 (22)	3.3 (3)
Pregnancies	One	11.3 (21)	14.2 (19)	2.8 (2)
	Two	11.5 (14)	15.0 (12)	3.4 (2)
	Three	15.5 (15)	20.3 (14)	2.6 (1)
	Four or more	12.5 (18)	17.8 (18)	0.0 (0)
Deliveries	Nulliparous (0)	2.3 (2)	2.8 (2)	0.0 (0)
	Primiparous (1)	2.9 (3)	1.7 (1)	3.2 (2)
	Multiparous (2 or more)	2.6 (5)	2.3 (2)	2.1 (3)
Abortions	No	2.5 (8)	1.7 (3)	2.5 (5)
	Yes	3.6 (2)	5.1 (2)	0.0 (0)
Gestational anemia	No	14.4 (40)	14.7 (39)	2.2 (1)
	Yes	17.8 (21)	19.3 (21)	0.0 (0)
Malaria in the last year	No	9.6 (40)	12.2 (39)	0.7 (1)
	Yes	**19,3 (27)** ^b^**	**33.8 (23)** ^b^**	**4.6 (4)** ^c^*
Use of mosquito net	Yes	2.0 (5)	2.5 (3)	1.2 (2)
	No	4.3 (6)	3.0 (3)	4.6 (3)
Gestational malaria	No	7.0 (24)	10.4 (21)	1.6 (3)
	Yes	**27.3 (27)** ^b^**	**33.3 (25)** ^b^**	**6.9 (2)** ^c^*
Placental malaria	No	3.8 (13)	5.5 (11)	1.0 (2)
	Yes	**35.8 (39)** ^b^**	**41.4 (36)** ^b^**	**11.5 (3)** ^c^*
Low birth weight	No	14.4 (61)	19.5 (56)	2.6 (5)
	Yes	20.7 (6)	30.0 (6)	0.0 (0)
APGAR	Normal (>6)	2.6 (7)	2.2 (3)	2.1 (4)
	Abnormal (<7)	**20.0 (1)** ^c^*	**50.0 (1)** ^c^*	0.0 (0)

^a^ Denominator corresponds to the number of subjects in each subgroup or category (rows of the table) evaluated in umbilical cord or peripheral blood, the numerator is the number of subjects with CM in said subgroup

^b^ Pearson’s Chi-square test.

^c^ Fisher’s Exact test.

*p<0.05.

**p<0.01.

There were no cases of CM among the women with stillbirths. The general frequency of CM (as well as the separate analyzes between peripheral and cord blood) did not show an association with length at birth (Mann-Whitney U p = 0.927) or head circumference (Mann-Whitney U p = 0.142).

In the bivariate analysis, the presence of placental and gestational malaria, and having had malaria during the last year showed a strong association with CM; When performing the multivariate adjustment, only placental malaria retained its significance, indicating that the other associations were possibly the product of an effect modification (Table 4).

**Table 4:** Regression model for identification of confounding variables associated with congenital malaria.

Variables of the model	Crude OR (95%CI)	Adjusted OR (95%CI)
Placental malaria (Yes/No)	13.93 (7.06–27.46) **	12.40 (5.68–27.06)**
Gestational malaria (Yes/No)	5.02 (2.73–9.20) **	1.69 (0.81–3.51)
Malaria in the last year (Yes/No)	2.25 (1.33–3.84) **	1.45 (0.70–2.98)

*p<0.05.

**p<0.01.

OR: Odd ratios. 95%CI = 95% confidence interval.

**Note**. Two variables with a bivariate association with CM were not included in the model:

1. Birth weight: given that in the logistic model, continuous variables do not generate an interpretable result.

2. APGAR: since only one subject with this abnormal score presented CM, which affected the power of the logistic model.

## Discussion

In this study, a frequency of 12.2% (95%CI = 9.4–14.9) was found, which is lower than those reported in studies from Africa that used a similar case definition, that is, positive by TBS or PCR in peripheral or cord blood samples, in which prevalences between 18.6% and 56.8% were reported [7, 11]. It is also totally different from studies that evaluated both blood samples with TBS, which reported frequencies of 6.1% [9] and 26.5% [10], and also from the figures of a meta-analysis in which no cases were reported for the Colombian studies (although this figure of 0.0 is consistent with the TBS results of the current study) [13]. This contrast in figures accounts for Colombia’s epidemiological and parasitological peculiarities that, despite not registering the high figures of African studies, show that CM is a significant problem, just like in other endemic areas on different continents.

Compared to Colombian studies that tested umbilical cord samples with TBS, the current data are the same as other two previous studies that did not find cases with this test [17, 19], and are lower than the first investigations in the country about CM that registered frequencies of 2.7% [14], 2.4% [16] and 1.8% [15]. When analyzing the cases diagnosed with PCR in umbilical cord samples, the results of the current study are the same as the Campos’s study, which registered 13.1% of affected patients [16]; but they are lower than previous investigations that have indicated figures of 27.0% [17] and 29.2% [19]. Only the study of the Bardají’s group reported lower figures with 3.0% by PCR [18]; This last data could be supported by the fact that its sources of information were those traditionally used in the country as part of passive surveillance, which generally presents high underreporting, which constitutes a clear detection bias, as has been indicated by other studies [21].

It is essential to highlight the high frequency of submicroscopic CM in the indicated studies and in this investigation, in which it corresponded to 100% of the cases. This finding is a serious problem because this type of infection does not receive treatment due to not being detected by the Colombian control programs, which are based on TBS [22], and that the infection can progress to severe malaria [5, 6, 23, 24]. It should also be considered that TBS, compared to PCR, has low sensitivity, so it is necessary to use diagnostic tests that have demonstrated their excellent cost-effectiveness for gestational malaria [25]. Likewise, submicroscopic infections would not allow the elimination of malaria by allowing permanent transmission of the parasite [26].

At this point it is important to highlight that diagnosis molecular is the more relevant advance since the 2000s, for the programs of malaria elimination, being PCR the most widely used because its detection limit (<0.02 parasite/μL), high sensitivity and specificity; that led it to be the gold standard in surveillance programs and active malaria diagnosis in several countries. Specifically, qPCR complies with these properties, by having one set of primers to identified a highly conserved region of the 18SrRNA gene of *P*. *falciparum*, *P*. *vivax*, *P*. *malariae*, and *P*. *ovale*. Also, when comparing microscopy and qPCR, the concordance is less than 90%, but the clinical, molecular, and sequencing data, resolve discordances in favor of qPCR [27].

The frequency of CM was statistically higher in the detection in the umbilical cord blood (16.2%), compared to neonatal peripheral blood (2.2%), which questions the definition, management, and follow-up of the cases. For this reason, some authors consider that the reference standard for the diagnosis of CM should be the demonstration of the parasite in the peripheral blood of the newborn, since its detection in the cord may indicate non-active infection, which would have important repercussions when carrying out a pharmacological treatment [6, 28, 29], but this has not been proven. It is clear that in terms of public health, epidemiological surveillance, and management of CM control programs, the detection of the parasite in the cord or peripheral blood shows deficiencies in the control of transmission and presents obstacles to the elimination of the infection in key groups, like pregnant women and their children.

On the other hand, CM was higher among newborn with an Apgar of less than 7, and the weight in neonates with CM was less than that of those without infection, which coincides with what was reported in some texts that show the clinical link of CM with greater risk of neonatal morbidity and low birth weight [7]. However, it should be noted that in this study, there was an association with lower weight (as a continuous variable) but not with low birth weight (binary variable), which coincides with other studies [9, 11], meaning that there is lower weight at birth, but without reaching levels lower than 2500g. Furthermore, in relation to APGAR, the Hangi’s group found no association of this variable [9], unlike the current study. It is not clear whether the absence of these statistical relationships is the product of low statistical power, given that most studies in CM are designed to determine the magnitude of the problem and not its risk factors. For this reason, it is necessary to carry out more descriptive studies to generate hypotheses about the factors associated with CM.

The CM was statistically higher in newborn whose mothers had malaria in the last year, gestational and placental malaria, although only placental malaria was significant in the multivariate adjustment. These findings differ from previous studies that have documented other factors associated with CM such as low birth weight, anemia, preterm delivery [7], maternal parasitaemia, pregnancies, maternal age [9, 11], maternal hemoglobin level [11], and use of mosquito net [10]. In this sense, the current study only agrees with the research of Hangi et al., who reported the association with placental malaria [9].

Multiple investigations have reported the importance of the placenta in explaining the clinical picture of fetal, neonatal, and infant malaria, although all conclude that the role of this organ remains largely unknown. In this line of thought, several studies have indicated that in placental malaria, there is an accumulation of red blood cells infected with *Plasmodium* spp. in the intervillous space, with infiltration of inflammatory cells that alter the cytokine profile and the exchange of substances at the maternal-fetal interface, which can lead to fetal death, low birth weight, premature delivery, and short stature; all these in interaction with other variables such as the number of pregnancies, time of perinatal infection, and maternal age and parasite load [9, 30–34]. Although most of these investigations have focused on the pathogenesis of *P*. *falciparum*, studies on *P*. *vivax* have described its ability to cause maternal anemia, spontaneous abortion, low birth weight, and CM, attributable to the generation of a greater inflammatory response, with fewer parasitaemias but greater elimination of erythrocytes [35]. There are studies in the same Colombian endemic region that confirm the pathogenic capability of *P*. *vivax* in microscopic or submicroscopic infections to produce malaria in the mother, as well as in the placenta and the gestational product [17, 25–37].

Regarding placental malaria according to species (*P*. *falciparum* or *P*. *vivax*), two studies carried out in Colombia did not find differences in histopathological placental changes [37, 38]. Coinciding with other reports that did not find differences in the morphology of the villi when comparing infections with *P*. *falciparum* or *P*. *vivax*., both species reduce the size, perimeter, and vascularization of the villi, reducing the surface area available for the exchange of gases per villi [39].

Finally, previous studies have shown a low proportion of CM in highly endemic contexts and a high prevalence of gestational and placental malaria [13, 40], which would show some degree of protection of the gestational product by different mechanisms that require more significant research effort.

Among the limitations, it is highlighted that was not possible to apply a probability sampling given that, *stricto sensu*, there is no sampling frame of pregnant women; their number was even approximated based on the number of births, since Colombian statistics do not record the number of pregnant women exposed to malaria. Besides, the present study could not overcome the small number of maternal and neonatal clinical and epidemiological variables that have been examined in the Colombian studies (similar to what is presented in most studies with retrospective information). That is why it must be declared that there is a lack of knowledge of these variables and, worse, of the determination processes in which they are immersed in the CM, which is why more research is required in different endemic areas. However, an important achievement of this work was to have collected a very high number of neonatal samples to examine them for the presence of CM, which allows moving forward in the consolidation of the study of this entity in our environment. As far as we know, this number of samples has not been evaluated before in Colombia or any other part of the Americas. It is pertinent to highlight that the diagnostic procedures applied for CM (TBS and qPCR) were the same in the time window studied (2009–2020).

It is important to emphasize that CM is very relevant in endemic areas, and it presents severe consequences for the newborn. However, generally it is a diagnosis that can only be fulfilled under research conditions, because it is a diagnosis that is only suspected when the history of maternal exposure during pregnancy is known, and if at the time of delivery there are resources available for the collection of blood samples from the cord or the newborn. For this reason, it is urgent to increase the resources allocated to the active and timely search of cases, using diagnostic tests with high sensitivity and specificity.

## Conclusion

A high frequency of CM was found, and all cases were submicroscopic, associated with a history of malaria in the mother and lower birth weight. Because the control program in Colombia is based on TBS, it must be improved with the inclusion of other tests that allow the detection of submicroscopic CM. In addition, the program has other limitations such as do not have specific control actions for pregnant women, absence of an active search for cases, lack of registration of asymptomatic and submicroscopic cases, passive surveillance system with detection bias; to these factors must be added the low funding for research in this field, and the concomitance with problems of poverty and inequities in the care of pregnant women.

## Supporting information

S1 Data (XLSX)
